# Correction: Development and validation of the health segment classification of population encompassed within Singapore (HealthSCOPES) framework

**DOI:** 10.1371/journal.pone.0337925

**Published:** 2025-12-02

**Authors:** Ian Yi Han Ang, Nabilah Rahman, Shing Hei Wong, Sheryl Hui-Xian Ng, Kyle Xin Quan Tan, Ke Xin Eh, Zheng Jye Ling, Andrea Su En Lim, Kelvin Bryan Tan, Sue Anne Toh

In Tables 3 and 4, the label “ME” in the fourth column is incorrect. The fourth column label should be ME/OR. Please see the correct Tables 3 and 4 here.

In the Statistical analyses subsection of the Material and methods, there is an error in the last sentence of the second paragraph. The correct sentence is: The outcomes studied were (i) number of polyclinic visits, (ii) number of SOC visits, (iii) number of ED visits, (iv) number of inpatient admissions, (v) total inpatient bed days (BD), (vi) admission to intensive care unit (ICU), and (vii) mortality.

In the Demographics subsection of the Results, there is an error in the sixth sentence of the second paragraph. The correct sentence is: Although requiring financial assistance is not a classification criterion for segments [J], [K], and [L], more than 40% of the residents in the segments were beneficiaries of CHAS Blue.

In the Validation subsection of the Results, there is an error in the first sentence of the paragraph. The correct sentence is: Of the 4,671,465 residents segmented for 2016, 4,653,020 were alive in 2017.

**Funding**

**Table 3:** Marginal effects and odd ratios relating to 2017 healthcare utilization of 2016 HealthSCOPES broad groups residents.

Variable	Broad group	n	ME/OR	95% CI	P-value
No. of polyclinic visits	[A]-[C]	3,489,877	Reference		
	[D-[H]	1,078,655	2.04	2.03-2.04	<.001
	[I-[J]	31,469	2.21	2.17-2.24	<.001
	[K-[L]	53,019	2.12	2.09-2.14	<.001
No. of specialist outpatient clinic visits	[A]-[C]	3,489,877	Reference		
	[D-[H]	1,078,655	1.80	1.79-1.81	<.001
	[I-[J]	31,469	6.05	5.97-6.14	<.001
	[K-[L]	53,019	4.40	4.35-4.46	<.001
No. inpatient admissions	[A]-[C]	3,489,877	Reference		
	[D-[H]	1,078,655	0.15	0.15-0.15	<.001
	[I-[J]	31,469	1.16	1.14-1.18	<.001
	[K-[L]	53,019	1.07	1.05-1.08	<.001
Total inpatient bed days	[A]-[C]	3,489,877	Reference		
	[D-[H]	1,078,655	0.98	0.97-1.00	<.001
	[I-[J]	31,469	7.14	6.90-7.38	<.001
	[K-[L]	53,019	8.88	8.66-9.11	<.001
No. of emergency department visits	[A]-[C]	3,489,877	Reference		
	[D-[H]	1,078,655	0.18	0.17-0.18	<.001
	[I-[J]	31,469	1.12	1.10-1.14	<.001
	[K-[L]	53,019	1.03	1.01-1.04	<.001
ICU admission	[A]-[C]	3,489,877	Reference		
	[D-[H]	1,078,655	6.59	6.36-6.83	<.001
	[I-[J]	31,469	27.13	25.35-29.01	<.001
	[K-[L]	53,019	27.84	26.35-29.40	<.001

[A]-[C]: staying healthy; [D]-[H]: living well with illness; [I]-[J]: getting well; [K]-[L]: maximizing quality life years; CI: confidence interval; ME: marginal effect; OR: odds ratio; ICU: intensive care unit.

**Table 4:** Marginal effects and odd ratios relating to 2017 healthcare utilization of 2016 HealthSCOPES segmented residents.

Variable	Broad group	n	ME/OR	95% CI	P-value
No. of polyclinic visits	[A]	2,976,346	Reference		
	[B]	478,769	0.44	0.43-0.44	<.001
	[C]	34,762	0.31	0.30-0.33	<.001
	[D]	17,206	0.97	0.94-1.00	<.001
	[E]	348,933	0.94	0.94-0.95	<.001
	[F]	572,964	2.93	2.93-2.94	<.001
	[G]	114,634	1.72	1.70-1.73	<.001
	[H]	24,918	1.63	1.60-1.66	<.001
	[I]	5,231	1.03	0.98-1.08	<.001
	[J]	26,238	2.52	2.48-2.55	<.001
	[K]	37,539	1.97	1.94-2.00	<.001
	[L]	15,480	2.69	2.64-2.74	<.001
No. of specialist outpatient clinic visits	[A]	2,976,346	Reference		
	[B]	478,769	0.18	0.17-0.18	<.001
	[C]	34,762	0.69	0.66-0.72	<.001
	[D]	17,206	1.20	1.16-1.23	<.001
	[E]	348,933	0.91	0.90-0.92	<.001
	[F]	572,964	2.02	2.01-2.03	<.001
	[G]	114,634	2.39	2.36-2.41	<.001
	[H]	24,918	8.36	8.24-8.49	<.001
	[I]	5,231	5.24	5.07-5.42	<.001
	[J]	26,238	6.25	6.16-6.35	<.001
	[K]	37,539	3.58	3.53-3.64	<.001
	[L]	15,480	6.50	6.37-6.63	<.001
No. inpatient admissions	[A]	2,976,346	Reference		
	[B]	478,769	0.02	0.02-0.02	<.001
	[C]	34,762	0.07	0.06-0.07	<.001
	[D]	17,206	0.12	0.11-0.13	<.001
	[E]	348,933	0.07	0.07-0.07	<.001
	[F]	572,964	0.19	0.19-0.19	<.001
	[G]	114,634	0.17	0.17-0.18	<.001
	[H]	24,918	0.54	0.52-0.55	<.001
	[I]	5,231	0.78	0.74-0.82	<.001
	[J]	26,238	1.24	1.22-1.26	<.001
	[K]	37,539	0.84	0.82-0.85	<.001
	[L]	15,480	1.64	1.61-1.67	<.001
Total inpatient bed days	[A]	2,976,346	Reference		
	[B]	478,769	0.11	0.10-0.12	<.001
	[C]	34,762	0.75	0.68-0.82	<.001
	[D]	17,206	0.41	0.36-0.45	<.001
	[E]	348,933	0.33	0.32-0.34	<.001
	[F]	572,964	1.19	1.17-1.21	<.001
	[G]	114,634	1.71	1.66-1.77	<.001
	[H]	24,918	3.43	3.27-3.60	<.001
	[I]	5,231	2.87	2.58-3.16	<.001
	[J]	26,238	8.02	7.74-8.30	<.001
	[K]	37,539	7.75	7.51-7.99	<.001
	[L]	15,480	11.69	11.22-12.17	<.001
No. of emergency department visits	[A]	2,976,346	Reference		
	[B]	478,769	0.07	0.07-0.07	<.001
	[C]	34,762	0.05	0.05-0.06	<.001
	[D]	17,206	0.20	0.19-0.21	<.001
	[E]	348,933	0.11	0.10-0.11	<.001
	[F]	572,964	0.21	0.20-0.21	<.001
	[G]	114,634	0.29	0.29-0.30	<.001
	[H]	24,918	0.32	0.31-0.33	<.001
	[I]	5,231	0.66	0.63-0.70	<.001
	[J]	26,238	1.22	1.20-1.25	<.001
	[K]	37,539	0.88	0.86-0.90	<.001
	[L]	15,480	1.42	1.38-1.45	<.001
ICU admission	[A]	2,976,346	Reference		
	[B]	478,769	1.42	1.32-1.54	<.001
	[C]	34,762	5.87	5.13-6.68	<.001
	[D]	17,206	2.73	2.07-3.53	<.001
	[E]	348,933	3.77	3.55-4.00	<.001
	[F]	572,964	9.35	8.97-9.75	<.001
	[G]	114,634	5.27	4.85-5.71	<.001
	[H]	24,918	22.29	20.45-24.26	<.001
	[I]	5,231	14.90	11.98-18.28	<.001
	[J]	26,238	33.09	30.78-35.54	<.001
	[K]	37,539	15.75	14.49-17.09	<.001
	[L]	15,480	69.59	64.98-74.47	<.001

[A]: mostly healthy children, adults & seniors; [B]: at risk children & adults; [C]: at risk seniors; [D]: children with chronic condition; [E]: adults & seniors with simple chronic condition; [F]: adults & seniors with complex chronic condition; [G]: adults & seniors with mental health condition; [H]: adults & seniors with cancer; [I]: children with transitional & long-term care needs; [J]: adults & seniors with transitional care needs; [K]: frail adults & seniors; [L]: end-of-life; CI: confidence interval; ME: marginal effect; OR: odds ratio; ICU: intensive care unit.
